# Integrin-Mediated Host Cell Invasion by Type 1–Piliated Uropathogenic Escherichia coli

**DOI:** 10.1371/journal.ppat.0030100

**Published:** 2007-07-13

**Authors:** Danelle S Eto, Tiffani A Jones, Jamie L Sundsbak, Matthew A Mulvey

**Affiliations:** Division of Cell Biology and Immunology, Pathology Department, University of Utah, Salt Lake City, Utah, United States of America; Tufts University School of Medicine, United States of America

## Abstract

Uropathogenic Escherichia coli (UPEC), the primary causative agent of urinary tract infections, typically express filamentous adhesive organelles called type 1 pili that mediate both bacterial attachment to and invasion of bladder urothelial cells. Several host proteins have previously been identified as receptors for type 1 pili, but none have been conclusively shown to promote UPEC entry into host bladder cells. Using overlay assays with FimH, the purified type 1 pilus adhesin, and mass spectroscopy, we have identified β1 and α3 integrins as key host receptors for UPEC. FimH recognizes N-linked oligosaccharides on these receptors, which are expressed throughout the urothelium. In a bladder cell culture system, β1 and α3 integrin receptors co-localize with invading type 1–piliated bacteria and F-actin. FimH-mediated bacterial invasion of host bladder cells is inhibited by β1 and α3 integrin–specific antibodies and by disruption of the β1 integrin gene in the GD25 fibroblast cell line. Phosphorylation site mutations within the cytoplasmic tail of β1 integrin that alter integrin signaling also variably affect UPEC entry into host cells, by either attenuating or boosting invasion frequencies. Furthermore, focal adhesion and Src family kinases, which propagate integrin-linked signaling and downstream cytoskeletal rearrangements, are shown to be required for FimH-dependent bacterial invasion of target host cells. Cumulatively, these results indicate that β1 and α3 integrins are functionally important receptors for type 1 pili–expressing bacteria within the urinary tract and possibly at other sites within the host.

## Author Summary

Strains of bacteria called uropathogenic Escherichia coli (UPEC) are the primary cause of urinary tract infections (UTIs), which by some estimates are the second most common type of infectious disease in the world today. UPEC strains typically express hair-like fibers called type 1 pili on their surface that allow them to bind and invade the host cells that line the urinary tract. The ability of UPEC to enter these host cells likely promotes the establishment and persistence of UTIs. The invasion process requires that the incoming pathogens first bind specific receptor molecules on the target cell surface. Here, we identify two host proteins known as β1 and α3 integrin as key receptors for type 1 pili–expressing UPEC. The adhesive tips of type 1 pili recognize sugars that decorate these integrin receptors, thereby activating a signaling cascade that stimulates the host plasma membrane to zipper around and envelop bound bacteria. β1 and α3 integrins have important roles in a number of host cell functions and are widely distributed within the urinary tract and in other tissues. Interestingly, type 1 pili are expressed by many different types of bacteria in addition to UPEC, raising the possibility that interactions between type 1 pili and integrin receptors can facilitate bacterial colonization within diverse niches throughout the host.

## Introduction

Host cell invasion contributes to the ability of many bacterial pathogens to colonize, multiply, disseminate, and, in some cases, persist for weeks to months within their animal hosts. In recent years, many bacteria that had previously been characterized as strictly extracellular microbes have been shown to behave as opportunistic intracellular pathogens [1]. Among these invasive bacteria are strains of uropathogenic Escherichia coli (UPEC). Worldwide, UPEC accounts for the majority of urinary tract infections (UTIs), including both cystitis (bladder infection) and pyelonephritis (kidney infection) [2]. These infections are exceedingly common in females, affecting about 11% of women each year [3]. The propensity of these infections to recur adds greatly to their problematic nature. By some estimates, more than 25% of women will endure a second UTI within 6 mo of an initial bladder infection, and about 3% will experience a third [4,5]. Cell culture and mouse UTI model systems suggest that UPEC invasion of host tissues within the urinary tract contributes significantly to the establishment as well the persistence and recurrence of UTIs [6–13].

UPEC are now known to utilize a number of diverse mechanisms to enter host epithelial cells [1,14]. These mechanisms include manipulation of host Rho GTPases by the secreted UPEC-associated toxin CNF1 [15], the hijacking, via opsonization, of host complement receptors [16,17], and the expression by UPEC of filamentous adhesive organelles known as type 1 pili [18]. Virtually all UPEC isolates and many other species within the Enterobacteriaceae family encode type 1 pili (also known as type 1 fimbriae) [19–21]. These peritrichously expressed organelles are composite fibers made up of a 7-nm-wide helical rod comprised of repeating FimA subunits joined to a distal 3-nm-thick tip fibrillum consisting of two adaptor proteins, FimF and FimG, and the adhesin FimH [22,23]. Type 1 pili, and in particular the FimH adhesin, are critical to the ability of UPEC to effectively colonize the urinary tract [8,19,24,25].

FimH possesses an N-terminal carbohydrate-binding pocket that enables type 1–piliated E. coli to bind mannose-containing host glycoprotein receptors and thereby mediate both bacterial adherence to and invasion of target host cells [18,26]. Studies using *fimH*-null mutants and purified FimH have demonstrated that this adhesin is sufficient for mediating E. coli entry into bladder epithelial cells [18]. Once internalized, UPEC is trafficked into membrane-bound acidic compartments with many features that are characteristic of late endosomes and lysosomes [10,18]. Within these compartments, UPEC replication is restricted. However, upon entry into the host cytosol, UPEC can rapidly multiply, forming large biofilm-like inclusions that occupy much of the host cell interior [6,9,10,27]. These inclusions are known variably as bacterial factories, pods, or intracellular bacterial communities and are often observed in mouse UTI models within the terminally differentiated superficial umbrella cells that line the lumenal surface of the bladder. Umbrella cells, along with two to three layers of underlying immature basal and intermediate epithelial cells, comprise the urothelium.

The stratified cell layers of the urothelium act as a formidable permeability barrier, restricting the diffusion of solutes from urine into associated tissue [28]. However, during the course of a UTI, this barrier is compromised due to the induced exfoliation of infected superficial umbrella cells and the influx of immune cells [1,8,13]. Consequently, UPEC is able to penetrate beyond the superficial cell layer and invade underlying immature host cells [9,10]. UPEC growth within these epithelial cells is limited, and the bacteria seem to enter a more quiescent state that is in part modulated by the host actin cytoskeleton. We have hypothesized that UPEC that manage to invade and persist quiescently within the immature cells of the bladder urothelium act as a source for recurrent acute UTIs [10].

How FimH promotes UPEC invasion of bladder epithelial cells is not entirely clear. Cell culture studies have revealed that FimH-mediated host cell invasion requires phosphoinositide 3-kinase (PI 3-kinase) activation and localized host actin rearrangements [18]. Rho GTPases and lipid rafts are also implicated in the invasion process, along with focal adhesion kinase (FAK) activation and transient complex formation between the cytoskeleton stabilizing and scaffolding components α-actinin and vinculin [18,29,30]. However, the FimH host receptor(s) involved in initiating the cascade of signaling events leading to UPEC entry into bladder epithelial cells is unknown. FimH interactions with several glycosylated and nonglycosylated host factors have been documented. These include components of the glycocalyx that sparsely covers the bladder surface [31], a few extracellular matrix (ECM) proteins [32–34], carcinoembryonic antigen-related cell adhesion molecule (CEACAM) family members [35,36], soluble Tamm-Horsfall protein [37], the glycosylphosphatidylinositol (GPI)-anchored protein CD48 [38,39], the leukocyte adhesion molecules CD11 and CD18 [40], and uroplakin 1a (UP1a) [41]. Here, we identify β1 and α3 integrins, which are distributed throughout the urothelium, as key mediators of host cell invasion by type 1–piliated E. coli. In addition, we show that FimH-mediated bacterial entry into host cells is modulated by specific phosphorylation sites within the cytoplasmic tail of β1 integrin and by downstream integrin-linked signaling factors.

## Results

### Identification of Potential FimH Receptors

To search for host receptors that might promote FimH-mediated bacterial invasion of bladder epithelial cells, we first purified the FimH adhesin. FimH is assembled into the distal tips of type 1 pili via a canonical chaperone-usher pathway [23,42]. In the absence of the periplasmic chaperone FimC, FimH, as well as other type 1 pilus subunits, misfold and are subsequently degraded. In order to purify native full-length FimH, the adhesin was coordinately expressed in BL21(DE3) E. coli cells with FimC, which was doubly tagged with consecutive COOH-terminal 6XHis and FLAG epitopes. These tags facilitated the purification of FimC–FimH complexes using affinity chromatography and allowed for cleaner detection of the complexes in later Far Western overlay assays.

Next, host membrane-associated proteins isolated from a human bladder epithelial cell line designated 5637 were resolved by SDS-PAGE and transferred to polyvinylidene fluoride (PVDF) membranes. These membranes were incubated with purified FimC_6XHisFLAG_ or with FimC_6XHisFLAG_–FimH complexes in the presence or absence of 2.5% D-mannose, which acts as a soluble FimH receptor analog. All blots were subsequently probed using an anti-FLAG antibody and chemiluminescence. In these Far Western overlay assays, no bands were bound by purified FimC_6XHisFLAG_ alone or by FimC_6XHisFLAG_–FimH complexes in the presence of D-mannose (Figure 1). However, in the absence of D-mannose, approximately 15 bands bound by FimC_6XHisFLAG_–FimH were detected. The mannose sensitivity of these interactions, as well as the lack of FimC_6XHisFLAG_ binding, indicates that they are mediated by FimH and not FimC. The two most prominent bands bound by FimH (Figure 1, bracketed) had molecular weights of approximately 120 and 150 kDa. These bands were excised from a duplicate GelCode Blue–stained gel, trypsin digested, and shown by mass spectroscopy to be composed of (in order of prevalence of the detected peptides) α2, β1, α3, α6, and β4 integrins. The presence of β4 integrin–specific sequences within these bands is surprising since this integrin has an apparent molecular weight of over 200 kDa, substantially above the range of the isolated 120- and 150-kDa bands. Nevertheless, we did not use this discrepancy as a basis to exclude β4 integrin as a FimH receptor candidate.

**Figure 1:**
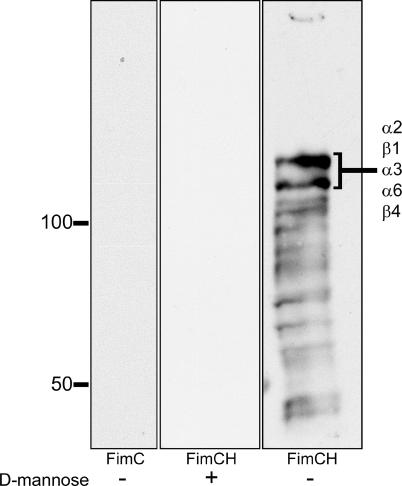
Identification of Potential FimH Receptors Membrane-associated proteins isolated from 5637 bladder epithelial cells were resolved by SDS-PAGE and transferred to PVDF membrane. Protein bands bound by purified recombinant FimC_6XHisFLAG_ alone (left) or FimC_6XHisFLAG_–FimH complexes in the presence (middle) and absence (right) of 2.5% D-mannose were detected by chemiluminescence in overlay assays using anti-FLAG antibody. The two most prominent bands bound by the FimC_6XHisFLAG_–FimH complexes were found by mass spectroscopy analysis to be comprised of α2, β1, α3, α6, and β4 integrins. Molecular weight standards are indicated on the left.

Integrins are surface adhesion molecules, comprised of α and β subunit heterodimers, that connect ECM proteins with the actin cytoskeleton [43]. Currently, there are 18 known α and eight β subunits that can combine to form at least 24 different heterodimeric integrin complexes with varying ligand-binding specificities. β subunits, and in particular β1, can interact with multiple α subunits. Many pathogens, including human cytomegalovirus, Group A Streptococcus, Yersinia spp., and AfaD^+^ *E. coli,* gain entry into target host cells by binding integrins either directly or indirectly via matrix proteins such as fibronectin [44–50]. Our data from Far Western overlay assays, which were performed in the absence of any established integrin ligands, suggest that FimH is able to directly interact in a mannose-sensitive fashion with one or more of the five integrin subunits identified above.

### Antibodies against β1 and α3 Integrins Inhibit Host Cell Invasion by UPEC

To test the functional role of the putative FimH receptors (β1, β4, α2, α3, and α6 integrins) in mediating UPEC entry into host cells, we employed a modified gentamicin protection–based invasion assay (see Materials and Methods and [51]). 5637 bladder epithelial cells were incubated with monoclonal antibodies directed against specific integrin subunits prior to infection with either UTI89, a well-characterized human cystitis isolate [6,9,10,27,52], or AAEC185/pSH2, a type 1 pili–expressing recombinant laboratory K-12 E. coli strain [8,18]. Both UTI89 and AAEC185/pSH2 require expression of the FimH adhesin in order to adhere to and effectively invade bladder epithelial cells. We found that a β1 integrin–blocking antibody, 6S6, notably decreased UTI89 invasion of host 5637 bladder epithelial cells (Figure 2A). The β1 integrin–activating antibody P4G11 and the anti–α3 integrin antibody P1B5 had similar effects in inhibiting bacterial invasion. Antibodies against the other putative FimH receptors (α2, α6, and β4 integrins) did not significantly affect UTI89 entry into the bladder epithelial cells and served as convenient isotype controls. Similar invasion results were observed in complementary experiments using AAEC185/pSH2. Both the β1 integrin–blocking antibody 6S6 and the anti–α3 integrin antibody P1B5 notably attenuated AAEC185/pSH2 invasion of 5637 cells, while an anti–α2 integrin control antibody had no effect (Figure 2C). None of the antibodies had any significant effect on the total numbers of cell-associated bacteria, with the exception of the anti–α3 integrin antibody, which for unknown reasons caused a slight increase in the numbers of cell-associated AAEC185/pSH2 (Figure 2B and 2D). Increasing the concentration of the antibodies used by up to 8-fold gave similar results in both the invasion and adherence assays. Importantly, control studies showed that none of the antibodies utilized in these assays cross-reacted or interfered with either the growth or viability of UTI89 and AAEC185/pSH2. The combined use of anti–α3 and anti–β1 integrin antibodies did not have a cumulative inhibitory effect on UTI89 invasion (unpublished data).

**Figure 2:**
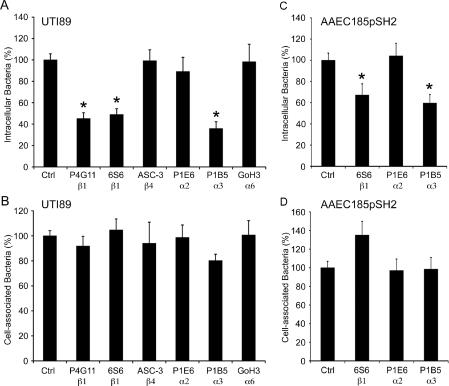
Anti–β1 and Anti–α3 Integrin Antibodies Inhibit Host Cell Invasion by Type 1–Piliated E. coli 5637 bladder epithelial cell monolayers were treated with 1.5 μg/well of the indicated monoclonal antibodies for 30 min prior to infection with the UPEC cystitis isolate UTI89 or AAEC185/pSH2, a type 1 pili–expressing recombinant K-12 strain. Shown are (A and C) intracellular bacterial titers, determined using gentamicin protection assays, and (B and D) total cell-associated bacteria (including both intra- and extracellular bacteria). Data are expressed as the means ± standard error of the mean of at least three independent experiments carried out in triplicate. * *p* < 0.001, versus untreated control (Ctrl) values, as determined by Student's *t*-test.

### Localization of Type 1–Piliated E. coli with β1 and α3 Integrins

Immunofluorescence confocal microscopy was used to determine whether β1 and α3 integrin subunits localize in bladder epithelial cells with adherent and/or invading type 1 pili–expressing E. coli. 5637 bladder cells were infected for 30 min with either the cystitis isolate UTI89 or AAEC185/pSH2. Samples were stained using primary antibodies specific for the individual β1 and α3 subunits or for α3β1 heterodimeric complexes. Fluorescently tagged phalloidin was used to visualize F-actin. Analysis of single cross sections from confocal *z*-stacks revealed that β1 and α3 subunits, as well as α3β1 heterodimers and F-actin, co-localize around adherent and invading UTI89 (Figure 3A–3C). In addition, F-actin and α3β1 integrin heterodimers were also found in association with type 1–piliated bacteria that had already penetrated into the bladder epithelial cells, below the host cell surface (Figure 3D). Similar results were seen with AAEC185/pSH2-infected bladder cells (Figure 3E–3G). In contrast, bacterial co-localization with F-actin and the α3 and β1 integrin subunits was not detected in bladder cells infected with either of the *fimH*-null mutants *ΔfimH* UTI89 or AAEC185/pUT2002. These results suggest a scenario in which FimH-mediated clustering of α3β1 integrin receptors triggers localized actin rearrangements that lead to the envelopment and internalization of type 1–piliated E. coli.

**Figure 3:**
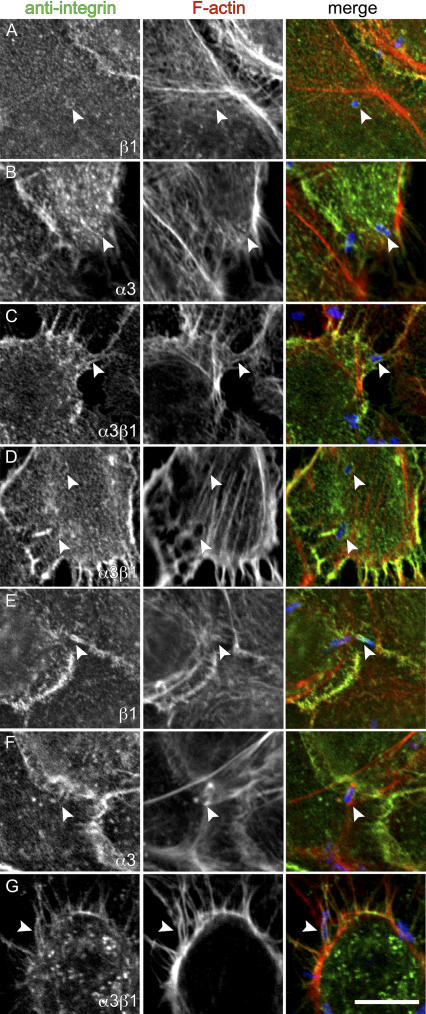
Localization of β1 and α3 Integrins with Type 1 Pili–Expressing E. coli 5637 bladder cells were infected for 30 min with (A–D) UTI89 or (E–G) AAEC185/pSH2 prior to fixation and processing for immunofluorescent confocal microscopy. Samples were stained using antibodies specific for the following individual integrin subunits: (A and E) β1, (B and F) α3, or (C, D and G) heterodimeric α3β1 integrin complexes (green in the merged color images). F-actin (red) was visualized using Alexa568-conjugated phalloidin, while bacteria (blue) were detected using anti–E. coli antibody. For clarity, the merged color image in each row is accompanied by images showing only corresponding single channel signals from integrin or F-actin staining. Arrowheads denote the location of an individual bacterium in each set of images. Based on optical sectioning, the highlighted bacteria in (A–C) and (E–G) were localized at or very near the host cell surface, while the bacteria in (D) have already completely penetrated the target host cell. Scale bar = 10 μm.

### Glycosidase Treatment of β1 and α3 Integrins Abrogates FimH Binding

The capacity of FimH to act as a lectin and bind mannose-containing structures is critical to its role as a mediator of bacterial adherence and invasion [18,53–55]. Our data suggest that FimH binds β1 and α3 integrin receptors directly, presumably via interactions with one or more of the N-linked sugar side chains that decorate these host glycoproteins. To further test this possibility, β1 and α3 integrins were immunoprecipitated from 5637 bladder cells that had been transfected with expression constructs encoding either V5His-tagged human β1 or untagged α3 integrin. Immunoprecipitates were split up and mock treated or treated with either of the glycosidases EndoHf or PNGase F. Samples were then resolved by SDS-PAGE and transferred to PVDF membranes. EndoHf cleaves high mannose and hybrid oligosaccharides from N-linked glycoproteins, while PNGase F cleaves all N-linked sugars. Western blot analysis using anti–β1 and anti–α3 integrin antibodies showed that the glycosidase treatments caused large decreases in the apparent molecular weights of both integrins, reflective of their deglycosylated status (Figure 4A and 4B). In Far Western overlay assays, purified FimC_6XHisFLAG_–FimH complexes interacted poorly or not at all with the deglycosylated forms of the β1 and α3 integrins, but bound well to the untreated integrin bands. Neither FimC_6XHisFLAG_–FimH complexes in the presence of 2.5% D-mannose nor FimC_6XHisFLAG_ alone bound to the untreated β1 and α3 integrin bands (Figure 4A and 4B). The faint bands seen in the Far Western blot shown in Figure 4B may reflect weak interactions between FimH and deglycosylated or partially glycosylated forms of α3 integrin—these faint bands were not detected in the presence of D-mannose or when blots were incubated with only purified FimC_6XHisFLAG_. Cumulatively, these results demonstrate that FimH can directly bind both β1 and α3 integrin subunits individually and that these interactions are highly dependent on the presence of N-linked oligosaccharides.

**Figure 4:**
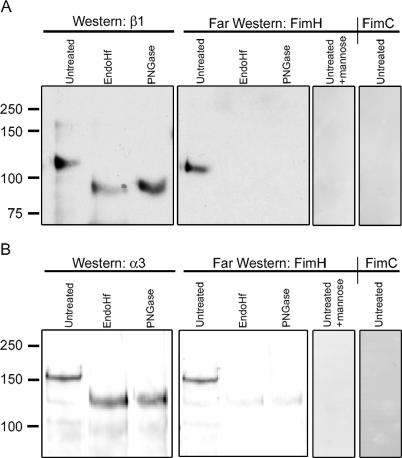
Glycosidase Treatment Abrogates In Vitro Binding of FimH to β1 and α3 Integrins (A) β1 and (B) α3 integrins were immunoprecipitated from 5637 cells that had been transiently transfected with plasmids for overexpression of recombinant human β1 or α3 integrins. The immunoprecipitated proteins were treated ± glycosidases (endoglycosidase Hf [EndoHf] or Peptide: N-glycosidase F [PNGase]) prior to SDS-PAGE and transfer to PVDF membranes. Blots were probed with either (A) anti–β1 or (B) anti–α3 integrin antibodies (Westerns), revealing expected shifts in the electrophoretic mobility of both integrin subunits following glycosidase treatments. For each set of samples, duplicate blots were overlaid with purified recombinant FimC_6XHisFLAG_–FimH complexes and probed with anti-FLAG tag antibody (Far Westerns). As additional controls, blots containing untreated β1 or α3 integrins were also incubated with either FimC_6XHisFLAG_–FimH complexes plus 2.5% D-mannose or with FimC_6XHisFLAG_ alone. Molecular weight standards are indicated on the left.

### Phosphorylation Sites within the Cytoplasmic Tail of β1 Integrin Modulate Entry of Type 1–Piliated E. coli

To confirm and further define the role of β1 integrin as a facilitator of host cell invasion by type 1–piliated *E. coli,* we performed cell association and gentamicin protection–based invasion assays with a mouse embryonic fibroblast cell line, GD25, in which the gene encoding β1 integrin has been inactivated [56,57]. Comparisons were made with a stably transfected derivative of the GD25 cell line, GD25-β1A, that encodes the widely expressed wild-type β1 integrin splice variant, β1A. In these and related assays described below, the number of intracellular bacteria recovered was divided by the number of total cell-associated bacteria as a means to normalize the data, accounting for possible variations in host cell numbers [51]. Entry of UTI89, as well as the type 1–piliated K-12 strain AAEC185/pSH2, into the β1 integrin–null GD25 cells was significantly decreased relative to the control GD25-β1A cells (Figure 5A and 5C). In contrast, the numbers of cell-associated bacteria did not notably differ between these two host cell lines (Figure 5B and 5D). These data verify that β1 integrin is a major, but probably not an exclusive, mediator of host cell invasion by type 1–piliated E. coli strains.

**Figure 5:**
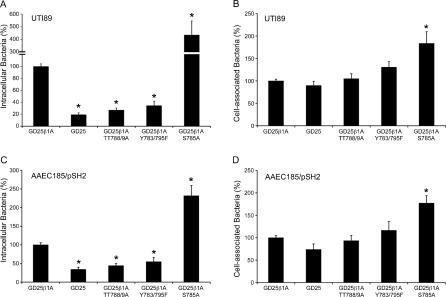
β1 Integrin Cytoplasmic Tail Mutants Differentially Affect FimH-Mediated Bacterial Invasion of Host Cells β1 integrin–null GD25 cells, as well as GD25 derivatives that constitutively express wild-type or the indicated mutant forms of β1 integrin, were infected with (A and B) UTI89 or (C and D) AAEC185/pSH2. Levels of intracellular bacteria (A and C) were normalized among the different host cell lines by dividing the numbers of intracellular, gentamicin-protected bacteria by the number of total cell-associated bacteria (B and D). Data are expressed relative to results from GD25-β1A control cells and represent the means ± standard error of the mean of at least five independent experiments performed in triplicate. * *p* < 0.001, versus values from control GD25-β1A cells, as determined by Student's *t*-test.

β1 integrin contains five potential phosphorylation sites (Y783, S785, T788, T789, and Y795) within the terminal 16 amino acids of its cytoplasmic tail [58]. Mutation of these phosphorylation sites can variably affect cellular attachment to ECM components as well as cell spreading and migration [58–60]. To address the roles of these phosphorylation sites within the cytoplasmic tail of β1 integrin in host cell invasion by type 1–piliated *E. coli,* we utilized three derivatives of the GD25 cell line: GD25-β1A_TT788/9A_, GD25-β1A_Y783/795F_, and GD25-β1A_S785A_ cells. GD25-β1A_TT788/9A_ cells have T to A mutations at threonine residues T788 and T789 within the cytoplasmic tail of β1A integrin that render the ligand-binding extracellular domain of this integrin inactive and unable to mediate cellular adhesion to laminin and fibronectin [58,59]. In GD25-β1A_Y783/795F_ cells, tyrosine residues Y783 and Y795 of β1A integrin have been replaced with phenylalanines, resulting in decreased cell migration presumptively due to reduced activation of FAK, a key regulator of focal adhesion and actin dynamics [61]. In GD25-β1A_S785A_ cells, serine residue S785 within the tail of β1A integrin was mutated to alanine. While no glaring phenotypic effect due to this particular amino acid substitution has been reported yet, other mutations at this site indicate a role for S785 phosphorylation as a modulator of cell attachment, spreading, and migration [60].

Results from invasion assays using GD25-β1A_TT788/9A_, GD25-β1A_Y783/795F_, and GD25-β1A_S785A_ cells infected with either UTI89 or AAEC185/pSH2 are included as part of Figure 5A and 5C. Similar trends were observed for both UTI89- and AAEC185/pSH2-infected samples. Relative to the GD25-β1A cells, bacterial entry into both GD25-β1A_Y783/795F_ and GD25-β1A_TT788/9A_ cells was significantly reduced. In contrast, bacterial invasion of GD25-β1_S785A_ host cells was notably and reproducibly enhanced. The numbers of total bacteria that could associate with these different host cell lines were similar, with the exception of the GD25-β1_S785A_ cells (Figure 5B and 5D). On average, nearly twice as many type 1–piliated bacteria associated with the GD25-β1_S785A_ cells in comparison with the parent GD25 cells.

Flow cytometry analysis confirmed that the GD25 cell line lacked β1 integrin surface expression and also revealed some variation in the levels of β1 integrin surface expression among the different GD25 derivatives (Table 1). Higher levels of β1 integrin expression on the surface of GD25-β1_S785A_ cells may contribute to their relative enhanced capacity to bind and internalize type 1–piliated bacteria. However, β1 integrin expression levels did not necessarily correlate with the ability of the bacteria to either associate with or invade the other GD25 cell line derivatives (Figure 5 and Table 1). For example, in our experiments, GD25-β1A_Y783/795F_ cells consistently expressed higher levels of surface-localized β1 integrin than GD25-β1_S785A_ cells, yet the GD25-β1A_Y783/795F_ cells were bound by fewer bacteria and were considerably less susceptible to invasion by type 1 pili–expressing E. coli. Cumulatively, these data indicate that phosphorylation site-specific mutations within the β1 integrin cytoplasmic tail can differentially affect host cell invasion by type 1–piliated E. coli strains. However, these effects are not entirely attributable to variances in receptor availability or bacterial adhesion frequencies and may instead be a consequence of aberrant integrin-linked signaling events.

**Table 1:**
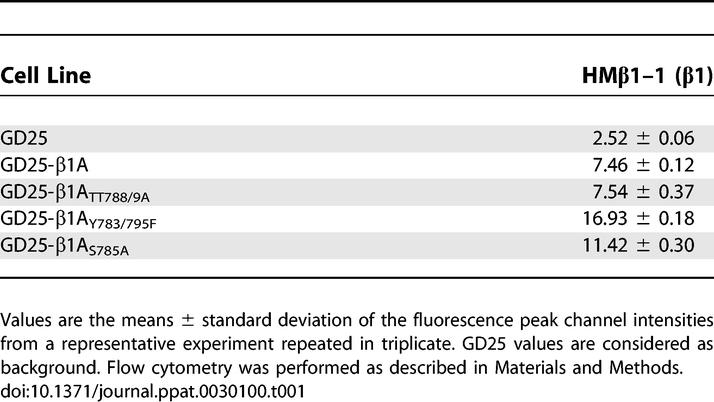
β1 Integrin Expression as Determined by Flow Cytometry

### Host Cell Invasion by Type 1–Piliated E. coli Requires Src and FAK

Ligand-induced clustering and activation of integrin receptors can stimulate a number of signal transduction pathways that often rely on Src family kinases and FAK [62,63]. The engagement of integrin receptors promotes autophosphorylation of FAK at tyrosine 397, creating a high-affinity binding site for several Src homology 2 (SH2) domain-containing proteins, including Src tyrosine kinase. Binding to FAK stimulates Src kinase activity, which in turn promotes further phosphorylation and maximal activation of FAK. The resultant dual-activated Src–FAK signaling complex acts to recruit and/or phosphorylate several adaptor and signaling factors that can modulate a variety of host cell functions, including actin dynamics. This link between integrin receptors and downstream signaling through Src family kinases and FAK suggested a role for these tyrosine kinases in FimH-mediated invasion of host cells by UPEC. To address this possibility, we first investigated the involvement of Src family kinases in UPEC invasion using two structurally related Src family kinase inhibitors, PP1 and PP2 [64]. Treatment with either PP1 or PP2 greatly inhibited the ability of UTI89 to invade 5637 bladder epithelial cells (Figure 6A). In contrast, treatment of host cells with PP3, an inactive analog of both PP1 and PP2, had almost no effect. Notably, these compounds did not interfere with bacterial association with the host cells (Figure 6B) and did not affect host cell or bacterial viability during the course of the experiments (unpublished data).

**Figure 6:**
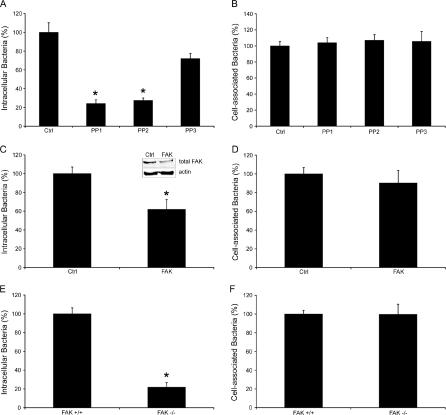
FimH-Mediated Bacterial Invasion of Host Cells Requires Src Family Kinases and FAK (A and B) 5637 cells were treated with 20 μg/ml of the Src family kinase inhibitors PP1 or PP2 or with an inactive analog, PP3, for 1 h prior to infection with UTI89. Levels of intracellular bacteria as determined by gentamicin protection assays (A) or total cell-associated bacteria (B) are expressed relative to results from cells treated with carrier (DMSO) alone. (C and D) Alternately, 5637 cells were transfected with either scrambled control siRNA or siRNA with specificity against FAK. Cells were infected with UTI89 at 72 h after siRNA transfection and intracellular (C) and total cell-associated (D) bacterial counts were determined. FAK knockdown was verified by Western blot analysis using antibody specific for total FAK ([C], inset). Blots were also probed using anti-actin antibody as a protein loading control. (E and F) Intracellular (E) and total cell-associated (F) bacteria were also quantified in assays using FAK−/− and control FAK+/+ mouse embryo fibroblasts. Intracellular bacterial levels were normalized in all assays by dividing the numbers of intracellular, gentamicin-protected bacteria by the number of total cell-associated bacteria. Data are expressed relative to results from the indicated control samples and represent the means ± standard error of the mean of three independent experiments performed in triplicate. * *p* < 0.001, versus values from control samples, as determined by Student's *t*-test.

To examine the role of FAK in host cell invasion by type 1–piliated UPEC, 5637 bladder cells were transfected with small interfering RNA (siRNA) specific for FAK or with control scrambled nonspecific siRNA. After 72 h, bacterial invasion and cell association assays were performed using the siRNA-transfected cells. FAK knockdown significantly reduced the ability of UTI89 to enter host 5637 cells by about 40% (Figure 6C), but did not affect the total numbers of host cell-associated bacteria (Figure 6D). Knockdown of FAK protein expression was verified by Western blot analysis, which indicated an approximately 65% reduction in FAK levels relative to levels of control samples that were transfected with nonspecific siRNA (Figure 6C, inset). To confirm the results from these siRNA experiments, we employed a mouse embryo fibroblast cell line that that does not express FAK (FAK−/− cells) [65]. FAK+/+ fibroblasts derived from littermates of the FAK−/− cell line progenitors were used as a control. UTI89 invasion of the FAK−/− cells was substantially inhibited relative to control cells (Figure 6E), while total numbers of cell-associated bacteria did not significantly vary between the two host cell lines (Figure 6F). Together, the results presented in Figure 6 demonstrate a requirement for both Src family kinases and FAK during FimH-mediated host cell invasion by UPEC.

## Discussion

In this study, we show that β1 and α3 integrins mediate entry of type 1–piliated E. coli into host cells. While other receptors for FimH have been previously identified, to our knowledge β1 and α3 integrins are the first demonstrated to be functionally important for UPEC invasion of uroepithelial cells. As a heterodimer, α3β1 integrin can bind a number of ECM components, including laminin, fibronectin, and collagen [66]. Recognition of ECM proteins allows many pathogens to interact indirectly with host integrin receptors [45]. FimH itself is able to bind the matrix-associated proteins laminin, fibronectin, and type IV collagens, which could in turn link UPEC with integrin receptors [32–34]. However, our results using Far Western overlay assays indicate that FimH can directly engage both β1 and α3 integrins, without the need for ECM components to bridge the interactions.

The carbohydrate-binding pocket at the distal tip of the adhesion domain of FimH accommodates α-linked mannose, either in free form or at the non-reducing end of a glycan [26,67]. Integrins can be differentially glycosylated depending on the cell line or cell type observed, and the number and types of glycosylations can affect integrin conformation and function [68–74]. Within their extracellular domains, the β1 and α3 integrin subunits have 12 and 13 putative N-linked glycosylation sites, respectively, and in the case of β1 integrin, ten of the 12 potential N-glycosylation sites appear to be utilized [75]. The N-linked glycans associated with both β1 and α3 subunits from different normal and cancerous urothelial cell lines are quite heterogeneous, with complex type oligosaccharides predominating over high-mannose type structures [72,76]. In the normal human urothelial cell line HCV29, the high-mannose glycans associated with both β1 and α3 subunits have terminally exposed mannose residues [76]. Although integrins with high-mannose type glycans constitute only a small fraction of the total integrin pool, the types of high-mannose structures present on this fraction are representative of those that are potentially able to interact with FimH. In particular, these high-mannose type glycans (Man_8_GlcNAc_2_ in both α3 and β1 integrin, along with Man_6_GlcNAc_2_ and Man_7_GlcNAc2 structures in β1 integrin) are similar to those that decorate an established FimH receptor, UP1a [77].

We have found that FimH binding to both α3 and β1 integrin subunits is competitively inhibited by the soluble receptor analog D-mannose, which suggests that the carbohydrate-binding pocket of the adhesin mediates FimH-integrin interactions. Furthermore, enzymatic deglycosylation of the integrin subunits abrogates interactions with FimH in overlay assays. Together, these results indicate that FimH recognizes one or more N-linked high-mannose oligosaccharides associated with each of the target integrins. The ability of FimH to bind the α3 and β1 subunits separately indicates that these interactions occur independently of the canonical ligand-binding pocket formed by intact α3β1 receptor heterodimers. Fine mapping of the interactions between FimH and the α3 and β1 integrin subunits awaits further studies.

The engagement of extracellular ligands induces integrin clustering, which in turn can enhance the ligand binding avidity of integrin receptors as well as activate downstream signaling events [43,78]. Previous work has already suggested that FimH-mediated invasion of bladder epithelial cells involves the induced clustering of host receptors [14,18]. By transmission electron microscopy, electron-dense material, which likely represents receptors and other host proteins, accumulates in the host membrane beneath adherent bacteria and FimH-coated beads as they are internalized. Results from immunofluorescence microscopy presented here indicate that α3β1 integrin heterodimers in bladder epithelial cells co-localize around adherent and invading type 1–piliated E. coli (see Figure 3). We have also noted the accumulation of F-actin around adherent and internalized bacteria in association with the integrin receptors.

F-actin dynamics are modulated downstream of integrin clustering by the recruitment and/or activation of a number of adaptor proteins, Rho family GTPases and kinases [79]. Several of these factors have been implicated as regulators of host cell invasion by type 1–piliated bacteria. These include the actin adaptor and bundling components vinculin and α-actinin, the Rho GTPases Cdc42 and Rac1, PI 3-kinase, and FAK [18,29]. Infection of bladder epithelial cells with type 1–piliated E. coli stimulates phosphorylation of FAK at Y397 and promotes transient complex formation between FAK and PI 3-kinase [18]. Interactions with FAK activate PI 3-kinase and thereby elicit the generation of 3-phosphoinositide second messengers that can alter actin cytoskeletal dynamics via a number of direct and indirect mechanisms [80–82]. Pharmacological inhibition of PI 3-kinase activity using either wortmannin or LY294002 has been shown to greatly reduce invasion of 5637 bladder cells by type 1–piliated bacteria [18]. Here, using siRNA and a FAK-null cell line, we have demonstrated that FAK itself is also required for FimH-mediated entry of UPEC into host cells. Maximal activation of FAK is stimulated by interactions with Src family kinases [62,63]. We have found that inhibition of Src family kinases using either the PP1 or PP2 inhibitor significantly interferes with FimH-mediated invasion of host bladder cells by UPEC. In contrast, an inactive analog of these inhibitors had only a modest effect. It should be noted that, for unknown reasons, a previous study using only the PP1 inhibitor failed to indicate a role for Src family kinases in FimH-mediated bacterial invasion [18].

FAK positively and negatively regulates multiple signaling molecules, including the Rho family GTPases Rac1 and Cdc42 [62]. Both of these GTPases appear to modulate FimH-dependent bacterial invasion of host bladder cells in possible conjunction with α-actinin and vinculin [29]. The adaptor protein α-actinin is itself a substrate for FAK, and the infection of bladder cells with type 1–piliated E. coli stimulates complex formation between α-actinin and vinculin [18]. These complexes can crosslink actin stress fibers and tether them to integrin receptor complexes via interactions with integrin-binding proteins such as paxillin and talin [63]. The recruitment of FAK and these various other host factors to the cytoplasmic tails of integrins appears to be temporally and spatially regulated by a number of different mechanisms, including phosphorylation. Mutational analyses of threonine, tyrosine, and serine residues within the cytoplasmic tail of β1 integrin indicate variable roles for these putative phosphorylation sites during host cell invasion by type 1–piliated E. coli.

Threonine residues T788 and T789 modulate the active conformation of the extracellular domain of β1 integrin via a so-called inside-out signaling process [58]. Inside-out signaling occurs when cytoplasmic signals confer a large conformational change to the integrin extracellular domain, converting the integrin from an inactive state to an active, high-affinity ligand-binding receptor [78]. The replacement of threonine residues T788 and T789 in the tail of β1 integrin with alanines alters the extracellular domain of the receptor so that it is inactive and can no longer bind fibronectin [58]. It is speculated that in wild-type cells, the phosphorylation of T788 and/or T789 is one means by which integrin inside-out signaling is regulated [58,83]. We have found that expression of the TT788/789AA mutant β1 integrin renders host cells refractory to invasion by type 1–piliated E. coli. Potentially, FimH interactions with β1 integrin in its activated state better promotes bacterial entry.

Tyrosine residues Y783 and Y795 within the cytoplasmic tail of β1 integrin may influence FimH-mediated bacterial invasion of host cells by altering both inside-out and outside-in signaling processes. Y783 and Y795 are part of two highly conserved NPxY motifs within the tail of β integrin subunits that act as specific binding sites for phosphotyrosine-binding domain-containing proteins, including β1 integrin cytoplasmic domain associated protein-1 and the focal adhesion components tensin and talin [84,85]. Binding of talin to NPxY motifs is thought to be required for inside-out activation of β integrins [86]. Mutation of the two tyrosines within the NPxY motifs of β1 integrin to alanines (Y783/795A) results in the complete loss of β1 integrin function [87,88]. In contrast, the replacement of these tyrosine residues with phenylalanines (Y783/795F), which partially mimic the hydrophobic aromatic ring structure of tyrosine and cannot be phosphorylated, preserves β1 integrin function during development in vivo [89,87]. These results indicate that the phosphorylation of Y783 and Y795 is not required for proper β1 integrin function under normal physiological conditions. However, earlier studies indicated that activation of FAK and cell migration is diminished in cultured cells that express β1A_Y783/795F_ integrin, which suggests that phosphorylation of Y783 and Y795 can alter integrin function in some situations [59,61]. In our assays, FimH-dependent bacterial invasion of GD25-β1A_Y783/795F_ host cells was significantly inhibited relative to control cells expressing wild-type β1 integrin. Similar results were previously reported for *Staphylococcus aureus,* a pathogen that invades host cells via α5β1 integrin receptors [90]. These findings contrast with those reported for *Yersinia* invasin-mediated entry into host cells via β1 integrin receptors, where the Y783/795F mutations were inconsequential [91]. The basis for these contrary outcomes is not clear, but they may reflect variations in the ways in which different bacterial pathogens engage and signal through integrin receptors.

In contrast to the T788/789A and Y783/795F mutations, the S785A mutation in the tail of β1 integrin significantly enhanced FimH-dependent bacterial invasion frequencies, which corresponded to a degree with increases in the number of total host cell–associated bacteria. Surface expression of β1 integrin in the GD25-β1A_S785A_ cell line was somewhat elevated relative to that of GD25-β1A control cells, and this could contribute to the observed effects. In addition, the S785A mutation may boost bacterial invasion frequencies by altering integrin-mediated signaling events. For example, it has been shown that mutations at S785 that mimic either de-phosphorylated or phosphorylated forms of serine differentially affect cell attachment, spreading, and migration [60]. Based on these observations, we suggest that S785 is an important regulator of β1 integrin signaling during FimH-mediated bacterial invasion, but the specific mechanism by which this residue affects the invasion process is not yet clear.

A critical feature of any host receptor is its location and availability on cells and within tissues that are targeted by incoming pathogens. As shown here, and by others [72,76], both β1 and α3 integrins are expressed by undifferentiated bladder epithelial cells. Furthermore, Southgate and colleagues have found that both β1 and α3 integrins are expressed in all strata of urothelia from the bladder, ureter, and renal pelvis [92]. Thus, β1 and α3 integrin subunits are well situated to act as receptors for UPEC entry into both the terminally differentiated superficial umbrella cells and the underlying immature cells of the urothelium. However, it is likely that other host proteins can also promote UPEC invasion of host urothelial cells. For example, in addition to β1 and α3 integrins, three other integrin subunits (α2, α6, and β4) are also prevalent within the normal human urothelium [92] and were picked up as potential FimH receptors in our initial screen. It is conceivable that one or more of these other integrin subunits may also facilitate FimH-mediated bacterial entry, but this was not demonstrable using available antibodies.

Due to its prevalence within the urinary tract, UP1a has received substantial attention as the canonical FimH receptor. Interestingly, UP1a is a member of a superfamily of membrane proteins known as tetraspanins that often complex with β1 integrin receptors [93]. It is thought that tetraspanins modulate integrin-dependent signaling as well as integrin trafficking and compartmentalization within lipid rafts on the cell surface. As already noted, FimH-mediated bacterial invasion of host bladder cells is apparently dependent upon lipid rafts [30]. Another tetraspanin protein, CD151, forms a particularly strong association with α3β1 receptors and potentiates integrin ligand-binding activity [94,95]. CD151/α3β1 integrin complexes interact with the complement receptor CD46 (membrane cofactor protein), a host protein that has recently been shown to enhance the efficacy of type 1 pilus–mediated bacterial invasion of human urinary tract epithelial cells [16,96]. These observations suggest the existence of an interconnected web of host receptors, co-receptors, and signaling molecules that can be recruited by type 1–piliated UPEC in order to facilitate its entry into target urothelial cells.

In addition to their roles as mediators of cell attachment, migration, and spreading, integrins are also important modulators of many other processes, including differentiation and apoptosis [43]. The capacity of FimH to bind α3β1 integrin, and perhaps other integrin subunits within the urothelium, raises the possibility that FimH effects extend beyond host cell adherence and invasion during the course of a UTI. For example, FimH interactions with α3β1 integrin at intercellular junctions could conceivably expedite bladder cell exfoliation and disruption of the urothelium barrier, a phenomenon that may promote UPEC dissemination within the urinary tract [1,8]. Furthermore, the recognition of α3β1 receptors by FimH may also modulate bacteria–host interactions outside the urinary tract. In addition to UPEC, most other extraintestinal pathogenic E. coli isolates and many other members of the Enterobacteriaceae family (including both commensal and pathogenic organisms) encode type 1 pili. These microbes are therefore potentially capable of binding and manipulating α3β1 integrin receptors that are expressed by a number of different host cell types and tissues within varied niches throughout the host.

## Materials and Methods

### Cell lines, bacterial strains, and plasmids.

The human bladder epithelial cell line 5637 (ATCC HTB-9; American Type Culture Collection, http://www.atcc.org/) was maintained in RPMI 1640 medium (Invitrogen, http://www.invitrogen.com/) supplemented with 10% heat inactivated fetal bovine serum (FBS) (HyClone, http://www.hyclone.com/) at 37 °C in 5% CO_2_. The β1 integrin–null cell line GD25 and its stably transfected β1 integrin–expressing derivatives GD25-β1A, GD25-β1A_S785A_, GD25-β1A_TT788/9A_, and GD25-β1A_Y783/795F_ (kindly provided by S. Johansson, The Biomedical Center, Uppsala, Sweden) were cultured in DMEM plus 10% FBS [56,57]. Puromycin (Sigma-Aldrich, http://www.sigmaaldrich.com/), at a concentration of 10 μg/ml, was used to maintain the stably transfected GD25 cell lines. The FAK-null mouse embryonic fibroblast cell line (FAK−/−; ATCC CRL-2644) and the control cell line (FAK+/+; ATCC CRL-2645) were maintained in DMEM plus 10% FBS. UTI89, a well-characterized human cystitis isolate, and the recombinant K-12 E. coli strains AAEC185/pSH2 (type 1 pili^+^) and AAEC185/pUT2002 (type 1 pili^+^/*ΔfimH* ), have been described [8,9,52]. Targeted deletion of *fimH* in UTI89 was accomplished by lambda Red–mediated linear transformation and verified by PCR as well as by yeast and red blood cell agglutination assays [97]. These strains were grown at 37 °C in 20 ml static Luria Bertani (LB) broth cultures for 48 h to stimulate expression of type 1 pili. pcDNA4A(β1_V5His6), which encodes the human β1A isoform with N-terminal V5 and 6XHis epitope tags, was generously provided by S. Kuwada (University of Utah, Salt Lake city, Utah, United States) [98]. pCMV_zeo_-hα3A, which encodes human α3 integrin, was provided by M. E. Hemler (Harvard Medical School, Boston, Massachusetts, United States) [99].

For coordinate expression and purification of FimC and FimH, we constructed a dual cistronic plasmid pJLJ200 encoding FimC with a COOH-terminal 6XHisFLAG-tag fusion and wild-type FimH under control of independent T7 promoters. *fimC* and *fimH* were amplified from plasmid pSH2 [100] using primers Cfor1 (5′-TGATACCATGGTGAGTAATAAAAACGTCAATG-3′), Crev2_HisFLAGN (5′-ACGTTAAGCTTATCCCTTATCGTCGTCATCCTTGTAGTCGTGGTGATGATGGTGATGTTCCATTACGCCCGTCATTT-3′), Hfor1 (5′-TTCAGCATATG AAACGAGTTATTACCCTGT-3′), and Hrev1 (5′-ACTTACTCGAGTTATTGATAAACAAAAGTCACGA-3′). The doubly underlined sequences indicate, respectively, NcoI, HindIII, NdeI, and XhoI sites that were introduced for cloning purposes. Singly underlined sequences indicate homology regions to the targeted *fimC* and *fimH* genes. Crev2_HisFLAGN was used to append sequences encoding adjacent 6XHis and FLAG tags at the COOH-terminus of FimC. Amplified *fimC_6XHisFLAG_* and *fimH* PCR products were digested using appropriate restriction enzymes and ligated, respectively, into multiple cloning sites 1 and 2 of pETDuet-1 (Novagen, http://www.emdbiosciences.com/html/NVG/home.html) to create pJLJ200. A construct encoding FimC_6XHisFLAG_ by itself was designated pJLJ300. For expression of recombinant proteins, the E. coli strain BL21(DE3) (Novagen) encoding isopropyl-1-thio-β-D-galactopyranoside (IPTG)-inducible T7 polymerase was transformed with either pJLJ200 or pJLJ300.

### FimC and FimCH purification.

BL21(DE3)/pJLJ200 was inoculated 1:100 from an overnight culture into 2 L of LB broth plus 100 ug of ampicillin/ml (Sigma-Aldrich) and grown shaking at 37 °C to a density of OD_600_ ∼0.6. At this point, expression of FimC_6XHisFLAG_ and FimH was induced by addition of 1 mM IPTG for 3 h. Bacteria were then pelleted, resuspended in cold 20 mM Tris (pH 8.0)/20% sucrose, and lysed on ice by addition of 5 μM EDTA and 75 μg of lysozyme/ml for 30 min. Next, 10 mM MgCl_2_ was added and unbroken bacteria and large debris were pelleted at 10,000*g* for 20 min at 4 °C. The supernatant, containing periplasmic proteins, was dialyzed against 20 mM Tris (pH 8.0) and loaded on Ni-NTA agarose columns (Qiagen, http://www.qiagen.com/). The column was washed with 20X column volumes of wash buffer (20 mM Tris [pH 8.0], 150 mM NaCl, and 20 mM imidazole) prior to elution of FimC_6XHisFLAG_–FimH complexes using 250 mM imidazole. Complexes were further purified using an anti-FLAG M2 agarose column (Sigma-Aldrich). Bound FimC_6XHisFLAG_ and associated FimH were washed with 15X column volumes of Tris-buffered saline (TBS, pH 7.4) prior to elution using 5 mg of FLAG peptide/ml (Sigma-Aldrich). Following the same protocol, FimC_6XHisFLAG_ in the absence of FimH was purified from strain BL21(DE3)/pJLJ300. The purity of the recovered recombinant proteins was determined by Western blot analysis and GelCode Blue–stained gels (Pierce, http://www.piercenet.com/).

### Overlay assays and identification of candidate receptors.

The membrane fraction from two confluent T75 flasks of 5637 bladder epithelial cell monolayers was isolated using an established protocol [101]. Briefly, cells were recovered by scraping into cold phosphate buffered saline (PBS), pelleted at 600*g* for 5 min, resuspended in 1 ml of HN buffer (5 mM HEPES [pH 7.5], 0.25 M sucrose) plus protease inhibitors (including 200 μM PMSF and a 1X protease inhibitor cocktail; Roche, http://www.roche.com/) and Dounce homogenized. Cell debris was removed by centrifugation at 600*g* for 10 min, and membranes in the resultant supernatant were pelleted by spinning at 10,000*g* for 30 min at 4 °C. The membrane pellet was lysed in 110 μl of 2X sample buffer (78 mM Tris [pH 6.8], 1% 2-mercaptoethanol, 0.001% bromophenol blue, 10% glycerol, 3% SDS) and heated for 5 min at 100 °C. Triplicate 20-μl aliquots, along with molecular weight standards in adjacent lanes, were resolved by SDS-PAGE using 12% polyacrylamide gels. Proteins from one set of lanes were stained using GelCode Blue while the other two sets were transferred to PVDF membrane and incubated for 20 min at room temperature in blocking buffer (TBS + 0.1% Tween-20 [TBS-T, pH 7.4], 1% BSA, and 1% powdered milk). Blocked membranes were then incubated for 90 min with 150 ug of purified recombinant FimC_6XHisFLAG_ alone or purified FimC_6XHisFLAG_–FimH complexes in 3 ml of blocking buffer +/− 2.5% D-mannose. Following 3 × 3-min washes with TBS-T, the membranes were incubated for 45 min with an anti-FLAG monoclonal antibody (diluted 1:1000; Sigma-Aldrich). After a final 30-min incubation with secondary anti-mouse IgG-HRP conjugate (1:10,000; Amersham Biosciences, http://www.gelifesciences.com/), blots were washed, developed using SuperSignal West Pico Chemiluminescent Substrate (Pierce), and exposed to CL-XPosure Film (Pierce). Host protein bands that bound purified FimC_6XHisFLAG_–FimH complexes, as determined by this overlay assay, were excised from the corresponding GelCode Blue–stained gel, subjected to in-gel trypsin digestion, and identified using liquid chromatography/mass spectrometry at the University of Utah core facility.

### Integrin immunoprecipitation and glycosidase treatments.

5637 cells at 80% confluency were transfected with pcDNA4A(β1_V5His6) or pCMV_zeo_-hα3A using the LT-1 transfection reagent (Mirus, http://www.mirusbio.com/). Then, 24 h post-transfection, 5637 monolayers were lysed in TNN buffer (50 mM Tris [pH 7.4], 150 mM NaCl, 1% NP-40, and 1X protease inhibitors) for 30 min on ice. Post-nuclear supernatants were pre-cleared with protein G-coupled agarose beads (Sigma-Aldrich) for 30 min at 4 °C prior to incubation with polyclonal anti-V5 tag (2 μg/ml; Bethyl Laboratories, http://www.bethyl.com/) or rabbit anti–α3 integrin antibody (5 μg/ml; Chemicon, http://www.chemicon.com/) for an additional 2 h at 4 °C. Immune complexes were precipitated using protein G-coupled beads and washed 4× with TNN lysis buffer. The beads were then divided into three samples and left untreated or treated with either 100 U of endoglycosidase Hf (EndoHf, 100,000 U/ml; New England Biolabs, http://www.neb.com/) or 5 U of Peptide: N-glycosidase F (PNGase F, 5,000 U/ml; New England Biolabs) for 60 min at 37 °C. Each sample was further divided in three and resolved by SDS-PAGE before transfer to PVDF membranes. The blots were overlaid with recombinant FimC_6XHisFLAG_–FimH complexes, FimC_6XHisFLAG_–FimH complexes plus 2.5% D-mannose, or FimC_6XHisFLAG_ alone and probed using anti-FLAG tag antibody as described above. The same blots were subsequently incubated with either polyclonal anti–β1 integrin antibody or rabbit anti–α3 integrin (both at 1:1000, Chemicon). Following incubations with HRP-tagged secondary antibodies (Amersham Biosciences), blots were washed and developed using SuperSignal West Pico Chemiluminescent Substrate (Pierce). Alternately, blots were incubated with IRDye-labeled secondary antibodies and visualized using an Odyssey Infrared Imaging System (LI-COR Biosciences, http://www.licor.com/).

### Invasion and cell association assays.

Bacterial invasion and cell association assays were performed essentially as previously described [18,51]. Within 24 h of seeding into 24-well plates, triplicate wells of confluent monolayers of host cells were infected with either UTI89 or AAEC185/pSH2 at a multiplicity of infection (MOI) of 1–15 bacteria per host cell. Plates were spun at 600*g* for 5 min to expedite and synchronize bacterial contact with the host cell monolayers prior to a 2-h incubation at 37 °C. For cell association assays, host cells were then washed 4× with PBS^2+^ (PBS supplemented with Mg^2+^/Ca^2+^) and lysed in 1 ml of PBS^2+^ containing 0.4% Triton X-100. For invasion assays, extracellular bacteria were killed following the initial 2-h incubation period by an additional 2-h incubation in medium containing the host membrane-impermeable bacteriocidal agent gentamicin (100 μg/ml; Sigma-Aldrich). Cells were then washed 4× with PBS^2+^ and lysed. Bacterial titers in the lysates were determined by serial dilutions and plating on LB agar plates. Except for the antibody blocking experiments, all results from invasion assays were normalized by dividing the number of intracellular bacteria by the total number of cell-associated bacteria [51]. Levels of intracellular and cell-associated bacteria were expressed as percentages relative to control samples, and all assays were repeated three or more times in triplicate. The significance of the observed differences was analyzed using Student's *t*-test (*p* < 0.05 was considered to be significant).

For the antibody blocking experiments, 5637 bladder cell monolayers in 24-well plates were pre-incubated with purified monoclonal antibodies for 30 min prior to the addition of bacteria. The final concentration of each antibody was 1.5 μg/well. Antibodies against human β1 (6S6), β4 (ASC-3), α2 (P1E6), and α3 (P1B5) integrins were purchased from Chemicon, while anti-human α6 integrin (GoH3) and the β1-activating antibody (P4G11) were obtained from R&D Systems (http://www.rndsystems.com/). The GoH3 antibody was also purchased from Abcam (http://www.abcam.com/). To test the role of Src family kinases on bacterial invasion, 5637 bladder cells were pre-incubated with PP1 (20 μM; Biomol, http://www.biomol.com/) or PP2 (20 μM; Calbiochem, http://www.emdbiosciences.com/html/CBC/home.html) for 1 h prior to infection. Control cells were treated with only the carrier (DMSO) or with PP3 (20 μM, Calbiochem). None of the inhibitors or DMSO alone had any effect on host cell or bacterial viability during the course of the experiments.

To knockdown FAK expression prior to invasion and host cell-association assays, 5637 cells were transfected with a FAK-specific siRNA SmartPool using Dharmafect 1 reagent (Dharmacon, http://www.dharmacon.com/). Control cells were transfected with scrambled siRNA. Invasion and cell association assays were performed 72 h after transfection. In parallel assays, FAK expression was assessed by Western blot analysis of transfected 5637 cell lysates prepared in TNN buffer plus protease inhibitors. Protein concentrations from post-nuclear supernatants were determined using the BCA reagent system (Pierce). Protein (20 μg) from each sample was resolved by SDS-PAGE and transferred to Immobilon-FL PVDF membranes (Millipore, http://www.millipore.com/). Following incubations with mouse anti-FAK (1:1000, BD Transduction Laboratories, http://www.bdbiosciences.com/) or mouse anti-actin antibodies (1:400, Abcam), blots were probed using IRDye-labeled secondary antibodies and visualized using an Odyssey Infrared Imaging System (LI-COR Biosciences).

### Flow cytometry analysis.

The surface expression of β1 integrin by the GD25 cell line and its derivatives was quantified by flow cytometry. Cells (1 × 10^6^) were incubated in PBS/2% FBS with a fluorescein isothiocyanate (FITC)-conjugated β1 integrin–specific antibody (HMβ1–1; Serotec, http://www.ab-direct.com/) or a FITC-labeled isotype control for 20 min on ice. After several washes, samples were assayed by FACScan analysis (Becton Dickinson, http://www.bd.com/). The mean fluorescence peak channel intensities reflect numbers of surface-localized β1 integrin receptors from HMβ1–1–stained samples. Signals from HMβ1–1–stained GD25 cells were comparable to those seen with samples treated with the isotype control antibody and is considered background. Three independent experiments were performed with similar trends in mean fluorescence intensities observed each time. However, the actual values differed depending on variations during the staining and acquisition setup for each experiment. Therefore, data is presented as the means +/− standard deviation from a single representative experiment repeated in triplicate.

### Immunofluorescence microscopy.

Thirty minutes post-infection with wild-type UTI89, *ΔfimH* UTI89, AAEC185/pSH2, or AAEC185/pUT2002 (MOI = 1–15), 5637 cells grown on 12-mm-diameter glass coverslips were washed with cold PBS^2+^ and fixed in acetone for 3 min at −20 °C. Samples were then rinsed 3 × 5 min with PBS, incubated for 30 min in blocking buffer (3% BSA/1% milk/PBS), and stained using rat anti-human β1 integrin (AIIB2, 1:100; Developmental Studies Hybridoma Bank at the University of Iowa, http://dshb.biology.uiowa.edu/), P1B5 (1:500; Chemicon), or mouse anti-α3β1 (1:250; Chemicon) and rabbit anti–E. coli (1:500; BioDesign, http://meridianlifescience.com/) primary antibodies along with appropriate Alexa Fluor-conjugated secondary antibodies (1:500; Molecular Probes/Invitrogen, http://probes.invitrogen.com/). Alexa568-conjugated phalloidin (1:40; Molecular Probes) was used to label F-actin. Following final washes in PBS, samples were mounted onto slides using FluorSave reagent (Calbiochem/EMD Biosciences) and viewed using an Olympus IX70 FV300 confocal microscope equipped with a 60× oil immersion objective (Olympus PlanApo NA 1.42 Oil immersion TIRFM), and argon and helium-neon (HeNe) lasers providing excitation energy at 488, 543, and 633 nm. Images were captured at the University of Utah School of Medicine Cell Imaging Facility using Olympus Fluoview software and subsequently analyzed using Volocity software (Improvision, http://www.improvision.com/).

## Supporting Information

### Accession Numbers

The GenBank (http://www.ncbi.nlm.nih.gov/Genbank/) accession numbers for the proteins discussed in this paper are α3 integrin (NP_002195), β1 integrin (NP_034708, NP_596867), FAK (AAA37592, AAA58469), FimC (P31697), FimH (AAX11338), and Src (NP_005408).

## Abbreviations

ECM: extracellular matrix
FAK: focal adhesion kinase
PI 3-kinase: phosphoinositide 3-kinase
PVDF: polyvinylidene fluoride
siRNA: small interfering RNA
UP1a: uroplakin 1a
UPEC: uropathogenic Escherichia coli
UTI: urinary tract infection
